# Transitioning of *Helicobacter pylori* Therapy from Trial and Error to Antimicrobial Stewardship

**DOI:** 10.3390/antibiotics9100671

**Published:** 2020-10-03

**Authors:** David Y. Graham

**Affiliations:** Department of Medicine, Michael E. DeBakey VA Medical Center and Baylor College of Medicine, RM 3A-318B (111D), 2002 Holcombe Boulevard, Houston, TX 77030, USA; dgraham@bcm.edu; Tel.: +713-795-0232; Fax: +713-795-4471

**Keywords:** *Helicobacter pylori*, antimicrobial stewardship, therapy, antibiotics, metronidazole, clarithromycin, fluoroquinolones, amoxicillin, proton pump inhibitors, ethical trials

## Abstract

*Helicobacter pylori* is the only major infection for which antimicrobial therapy is not designed using the principles of antimicrobial stewardship. Traditionally, antimicrobial therapy is a susceptibility-based therapy, achieves high cure rates, and includes surveillance programs to regularly provide updated data regarding resistance, outcomes, and treatment guidelines. Current *H. pylori* therapies identified by trial-and-error, and treatment recommendations and guidelines are based on comparisons among regimens that rarely take into account the prevalence or effect of resistance. The majority of patients currently treated achieve suboptimal results. A paradigm shift is required to abandon current approaches and embrace antimicrobial stewardship, and therefore reliably achieve high cure rates; develop, propagate, and update best practice guidelines; and provide surveillance of local or regional susceptibility/resistance patterns. These also require timely updates to clinicians regarding the current status of resistance, antimicrobial effectiveness, and ways to prevent antimicrobial misuse to extend the useful life of currently available antibiotics. Here, we discuss the differences among current approaches to *H. pylori* therapy and antimicrobial stewardship and identify what is required to achieve the transition. Conceptually, the differences are significant, and the transition will likely need to be both abrupt and complete. Recommendations for therapy during the transition period are given.

## 1. Introduction

The widespread misuse of antibiotics has resulted in increasing antimicrobial resistance which threatens the continued usefulness of currently available antimicrobials. The consequences of critical antibiotics becoming clinically ineffective have resulted in strong pressure to utilize the principles of antimicrobial stewardship for selecting and managing therapy for infectious diseases [1,2]. Management of *Helicobacter pylori* infections has long been within the purview of *Gastroenterology* which has been slow to accept the paradigm shift needed to change the approach to therapy from one utilizing trial-and-error to one based on the principles of antimicrobial stewardship. *H. pylori* was officially recognized as an infectious disease in 2015 [3], and those involved in studying *H. pylori* therapy are only now gradually beginning to accept that *H. pylori* therapy should no longer be exempt from the guidelines and practices governing treatment of other infectious diseases (i.e., the principles of antimicrobial stewardship).

## 2. Antimicrobial Stewardship in Traditional Infectious Disease Therapy

The principles of antimicrobial stewardship codified and extended thinking and practices involved in the development and implementation of methods to simultaneously improve antimicrobial therapy, prevent antimicrobial misuse, and reliably achieve high cure rates, while minimizing the risk of developing resistance in order to prolong the useful life of antibiotics. Until now, the approach to *H. pylori* therapy has not routinely involved elements critical to antimicrobial stewardship, such as optimization of the therapy in terms of drugs, dosing, or duration of therapy. As a result, current therapies have subsequently failed to reliably achieve high cure rates without prescribing unnecessary antimicrobials or prolonging therapy beyond the experimentally identified optimal duration. Stewardship elements that are also lacking include the development, propagation, and regular updating of best practice guidelines, as well as susceptibility/resistance surveillance to update clinicians of the current status of resistance and antimicrobial effectiveness, and the availability and potential role of new antimicrobials.

Fundamentally, a successful antimicrobial therapy is a susceptibility-based therapy, as success is predicated on the target organism being susceptible to the antimicrobial agents utilized. Antimicrobial therapies are also optimized so as to reliably achieve excellent results (i.e., high cure rates such as >95%) (Table 1) [4]. Comparisons among therapies are infrequent and, when done, compare proven highly effective regimens using noninferiority methodology [5,6,7]. As we discuss below, *H. pylori* therapy is most often prescribed empirically and most often fails to reliably achieve high cure rates [8]. Comparisons of empiric therapies are the rule and they focus on which is the better of the two regimens with seemingly little regard to the actual, often poor, cure rates achieved. In addition, consensus and guideline recommendations regarding *H. pylori* therapy rarely involve the principles of antimicrobial stewardship and are based on principles used for other gastrointestinal diseases, such as irritable bowel syndrome, which fail to reflect the marked differences and realities of antimicrobial therapy.

## 3. Original Development of *H. pylori* Antimicrobial Therapies

Soon after the discovery of *H. pylori*, it was shown that, in vitro, the organism was susceptible to a wide variety of commonly used antimicrobials. However, the infection proved to be difficult to cure using those antimicrobials, and it also proved impossible to reliably achieve high cure rates [9] (reviewed in [10]). This prompted a period of trial-and-error during which clinical trials were done with a wide variety of antimicrobials (reviewed in [10]). Eventually Tom Borody in Australia discovered an effective three-drug regimen consisting of bismuth, metronidazole, and tetracycline called bismuth triple therapy regimen [11].

*H. pylori* therapy was initially focused on treatment of peptic ulcer disease which was extremely common and one of the major gastrointestinal causes of morbidity, surgery, and medical costs. The basic approach was to treat the ulcer with a histamine-2 receptor antagonist (H2RA) and the infection with bismuth triple therapy [12]. It was soon recognized that the presence of metronidazole resistance greatly reduced the effectiveness of bismuth triple therapy, but that this could be partially or completely overcome by adding a proton pump inhibitor (PPI) and increasing both the dosage of metronidazole and the duration of therapy (e.g., to 14 days) (reviewed in [13]). This modified regimen is called bismuth quadruple therapy.

Bismuth triple therapy was commercially marketed in 1996 and while widely used with a PPI, the commercialization of bismuth quadruple therapy, in 2006, was delayed until after the patent regarding the addition of a PPI expired.

## 4. Development of *H. pylori* Therapies with Different Antibiotics

One of the initial attempts to produce an *H. pylori* therapy was with the macrolide, erythromycin. Erythromycin was able to suppress the infection but was unable to cure the infection [14]. Thus, macrolides were considered to be ineffective in vivo, until the introduction of clarithromycin which, when combined with a PPI or H2RA to increase gastric pH, proved effective. The initial dual therapy combination of clarithromycin and a PPI ultimately failed because of emergence of clarithromycin resistance during therapy [15,16,17]. However, with the addition of amoxicillin, it proved both well tolerated and effective [17]. This three-drug regime is called clarithromycin triple therapy or, alternatively, standard triple therapy. The effectiveness of clarithromycin triple therapy subsequently resulted in the development of additional triple therapies consisting of PPI and amoxicillin plus metronidazole, a fluoroquinolone, or rifabutin.

The original bismuth quadruple therapy was also modified by replacing the metronidazole with amoxicillin or furazolidone. Other recent successful iterations include bismuth and a PPI plus tetracycline and amoxicillin or metronidazole and amoxicillin (reviewed in [13,18,19]). The most recent advance has been with a dual therapy consisting of omeprazole plus amoxicillin. This was originally introduced in 1989 but proved unable to reliably achieve high cure rates and, until recently, was generally considered to be a failure [20,21,22] (reviewed in [19]). However, the introduction of potassium competitive acid blockers (P-CABs), such as vonoprazan, have reinvigorated interest in dual therapies (see below) [23].

## 5. The Effect of Gastroenterology Rather Than Infectious Disease Being Responsible for Development of *H. pylori* Therapies

Within a few years of discovery, the diagnosis and management of *H. pylori* had primarily become the responsibility of gastroenterology. Gastroenterologists found little need, or desire, to incorporate susceptibility testing as a key element required for choosing, defining, optimizing, and evaluating the results of *H. pylori* therapies. Instead, they adopted the trial-and-error approach which focused on comparison of therapies. This approach has remained the standard despite evidence that overall cure rates were often both poor and declining.

The gastroenterologist’s approach to the development of *H. pylori* therapies was most likely based on the long experience with common gastroenterology diseases, such as constipation or irritable bowel syndrome. Most diseases in gastroenterology are characterized as follows: (a) the cause is largely unknown, (b) there is a large placebo response to therapy, and (c) the success of most therapies is low requiring a comparator which is often a placebo. The fact that the etiologies were unknown required a focus on the results rather than an explanation for a poor response. This contrasts with infectious disease where the cause of a poor response is discoverable. These differences resulted in two different approaches, one that focused on outcome as a comparison (the what school) and the other that focused on the actual outcome (i.e., cure rate) (the why school). In gastroenterology, the “what school” has dominated planning, analysis, and reporting of clinical trials which can be best characterized as comparisons of arbitrary regimens focusing on the difference, despite the fact that often none of the arms achieved a high cure rate. This approach was abetted by medical journals which expected, even demanded, comparative trials. Eventually, thousands of patients were enrolled in *H. pylori* treatment trials of which many, if not most, achieved poor cure rates in at least one treatment arm. The fact that at least one arm in a trial achieved a poor result was, however, often not a result of chance. The published study design confirmed that based on prior experience, the authors reliably predicted which regime would produce the unacceptably low cure rate. By definition, informed consent requires this information to be shared with the subjects, but it was withheld [24,25]. Informed consent also requires informing patients about any new information arising during the trial and allowing them to reconsider. This also was not done. These issues with informed consent have continued to plague *H. pylori* comparative trials up to the present.

The development of sequential therapy, probably, best illustrates the differences between gastroenterology and the traditional development of therapy for an infectious disease. Sequential therapy is a regimen in which a dual therapy with a PPI and amoxicillin are given for five days, followed by five days with the PPI, clarithromycin, and metronidazole. It was developed in Italy and was prompted by increasing treatment failures with triple therapy. By 2001, it had been conclusively shown (e.g., studies involving more than 53,000 patients) that, because of increased clarithromycin resistance, clarithromycin triple therapy was no longer able to reliably achieve high cure rates [26,27]. Clarithromycin triple therapy was used as the straw man in multiple studies in which more than 1800 patients participated. The investigators were able to show that, in the mid-2000s, in one region of Italy, sequential therapy yielded higher cure rates than clarithromycin triple therapy [28]. The fact that prior trials in the same population had already proven that one regime was inferior, was also withheld from the consent of subjects in subsequent trials. Despite many iterations of the same trials, sequential therapy was neither optimized nor explored for its weaknesses. When sequential therapy was tested in different geographic areas with different resistance patterns (e.g., resistance to clarithromycin and metronidazole vs. increased resistance to clarithromycin but low resistance to metronidazole) sequential therapy proved ineffective and was abandoned [28,29].

The traditional infectious disease approach focuses on attainment of a prespecified cure rate (e.g., ≥95%) (i.e., the why school) using susceptibility-based therapy and would never have been included in the empiric comparisons described above. Attempts to optimize sequential therapy would have discovered that (a) the duration of therapy of 14 days provided a higher cure rate, (b) that sequential therapy was only effective in the presence of isolated clarithromycin resistance, and (c) that all those with clarithromycin resistance received the clarithromycin with no benefits. As noted previously, comparisons of highly effective susceptibility-based therapies are rare and, when preformed, are generally limited to head-to-head comparisons of proven, highly reliable, optimized regimens using noninferiority methods with both regimens expected to achieve high cure rates [7]. Typically, in science, observation (the what) is typically followed by experiments to understand the phenomenon (the why) (i.e., they are complimentary). *H. pylori* therapy has generally stopped with the what.

## 6. Meta-Analysis and *H. pylori* Therapy

Although meta-analysis has become the holy grail for analysis of studies in gastroenterology, it was often used inappropriately for assessing *H. pylori* therapy. The main problem has been that the comparisons involved were often fatally flawed. For example, if one compared 14-day sequential therapy with 14-day clarithromycin triple therapies in treatment of adherent patients with susceptible infections, one would expect both to have very similar high cure rates. Different patterns of resistance would be an obvious important difference (e.g., to clarithromycin and to metronidazole which is only present in one of the therapies), whereas relative potency of the PPI used is not (i.e., 40 mg of pantoprazole = 9 mg of omeprazole, whereas 40 mg of esomeprazole = 64 mg omeprazole [30,31]. If the populations did not differ in relation to resistance patterns or PPI relative potency, one would expect both to yield high cure rates. Failure to do so would signify the presence of important differences between the two populations such that they could not be compared as the results would be nongeneralizable and produce flawed conclusions. When meta-analyses compare trials where the data for each population is population specific and not generalizable, they are best described as Shmeta analyses [4,30]. Generalizability is one of the key requirements for valid and ethical research (Table 2) [32].

Another example of misuse of meta-analyses has been when it was used to provide guidance regarding therapy. For example, the 2017 American College of Gastroenterology guideline used a meta-analysis to show that bismuth quadruple therapy should replace triple therapy. They provided “an updated meta-analysis, which included 12 randomized controlled trials (RCTs) with 2753 patients; the intention-to-treat (ITT) eradication rate was 77.6% with bismuth quadruple therapy vs. 68.9% with clarithromycin triple therapy” [33]. However, because both regimens achieved clinically unacceptably low cure rates, the appropriate conclusion, based on the results presented, would be that neither should be used as an empiric therapy (at least in the regions where the studies were done).

Although *H. pylori* gastritis is an infectious disease of known cause for which reliably high cure rates are possible and there is no placebo response, the current status is that consensus statements and guidelines have been ineffective, and most patients continue to receive largely poor effective therapy [8,34].

## 7. The Role of Pharmaceutical Companies in Developing *H. pylori* Therapy

Pharma became involved with *H. pylori* when PPIs were new drugs and peptic ulcer was the major disease for which antisecretory drugs were used. H2RA’s were proven effective for treatment of peptic ulcers and omeprazole was having a difficult time becoming accepted. *H. pylori* represented an opportunity as it was a new problem without a simple and effective therapy. PPIs also appeared to possibly have a role to play in therapy and, in order to promote omeprazole, AstraZeneca sponsored a series of consensus conferences regarding *H. pylori* (including Maastricht I) [35,36]. This proved to be a win-win for AstraZeneca, as well as for spreading interest and knowledge regarding *H. pylori*. It also helped solidify *H. pylori* as a gastroenterology disease. Other pharmaceutical companies’ subsequent involvement primarily was to promote their anti-*H. pylori* therapies. The most recent example has been designed to promote the bismuth quadruple therapy, Pylera^®^, in Europe. Pharma’s goals included ensuring that their regimens, plus their suggested duration of therapy, were included in lectures, consensus conferences, and guidelines. Most regimens are now off-patent which has reduced, but not eliminated, pharmaceutal company influence on the knowledge and recommendations disseminated.

## 8. The Role of the U.S. Food and Drug Administration

**Figure 1:**
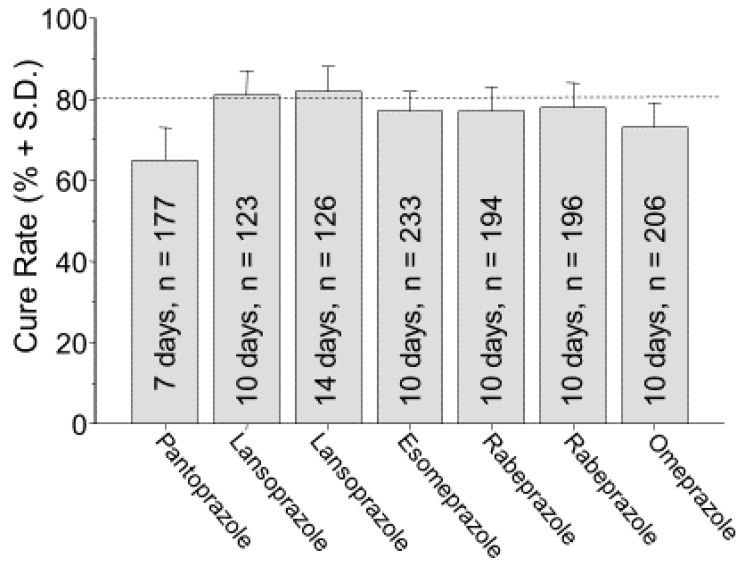
Intention to treat cure rates and standard deviations reported from the clinical trials published for studies designed to obtain FDA approval for triple therapy with PPI, clarithromycin, and amoxicillin, in the United States. From [4] with permission.

The original approvals of *H. pylori* therapies by the U.S. Food and Drug Administration (FDA) were obtained during the period when high cure rates could not be reliably achieved (Figure 1) [37,38,39,40]. Although it is a common misconception, FDA approval does not carry with it any implication that the regimen has been optimized in terms of doses or duration of therapy or that it will reliably achieve high cure rates. For example, the cure rates reported in the studies used to obtain approval of clarithromycin triple therapies ranged from 79% to 86%. The cure rates with clarithromycin triple therapies were the following: 77% with omeprazole (for 10 days), lansoprazole (for 10 and 14 days), rabeprazole (for 7 days); 78% with rabeprazole (for 10 days); and 83% with esomeprazole (for 10 days) [17,41,42,43] (Figure 1) [37,38,39,40]. The pivotal study with pantoprazole was not submitted for FDA approval, likely because the per protocol cure rates were relatively low (70% for the clarithromycin and 76% for the metronidazole seven-day triple therapy) [37]. Of interest, whenever two durations of therapy were examined (e.g., 7 and 10 days or 10 and 14 days), the shorter duration was always selected for the marketed version.

The original bismuth triple therapy (Helidac^®^) was approved using small studies that had been completed independently of the pharmaceutical company [44]. It was marketed for 14 days and could be used directly or with an H2RA. The more recent version as a three-in-one combination product (Pylera^®^) was studied and subsequently marketed for 10 days to offer a commercial advantage over the traditional product [45]. No comparisons were, or have been, done to address what is the optimum duration of therapy in the presence of metronidazole resistance (see below). The optimum duration must be defined experimentally rather than by marketing efforts of Pharma.

## 9. Basis for the General Recommendation for a Treatment Duration of 14 Days

*H. pylori* is one of those organisms, like *Mycobacterium tuberculosis,* that can enter a dormant state (persister state) in which its metabolism slows as does the need for replication. This process allows the organism to survive despite the presence of antibiotics [46,47,48]. As noted above, although therapy would appear to have eliminated the infection, early experiments found that, *H. pylori* was only suppressed and would rapidly reappear. Reappearance either denoted emergence of resistance or, if the organism remained susceptible to the antibiotics used, the duration of therapy was inadequate. The traditional response to recurrence without resistance is to lengthen the duration of therapy. With tuberculosis, this may require many months of therapy, with *H. pylori* 14 days appears to be sufficient. With *H. pylori,* this phenomenon is most often seen with amoxicillin-containing therapies. Because *H. pylori* only replicates within a narrow pH range (near pH 7), strategies to enhance killing would be to maintain an intragastric pH of greater than six, increasing the duration of therapy, or both [46,49,50].

## 10. Optimization: Duration of Therapy

The principles of antimicrobial stewardship require that therapies be optimized to achieve the highest cure rates while taking into consideration safety and cost-effectiveness. Because of the general observation regarding amoxicillin-containing regimens that 14-day therapy is generally superior to shorter durations, it has been recommended that the initial trial should be for 14 days and, only if the 14-day therapy proves highly effective, should one consider testing shorter durations [24]. The effectiveness of PPI plus amoxicillin-containing triple therapies is duration dependent. For example, when these triple therapies are given to patients with susceptible infections, the cure rate is typically between 88 and 92% with a 7-day regimen, 90–94% with a 10-day regimen, and 94–98% with a 14-day regimen [51,52]. PPI, amoxicillin, fluoroquinolone triple therapy is an excellent example of a marked delay in recognizing that it was possible to achieve high cure rates with this regimen. A possible bias by clinical investigators toward shorter durations resulted in a large number of studies and meta-analyses with PPI, amoxicillin, fluoroquinolone triple therapy [53] before it was recognized that cure rates ≥95% were obtainable with 14-day therapy [54].

**Figure 2:**
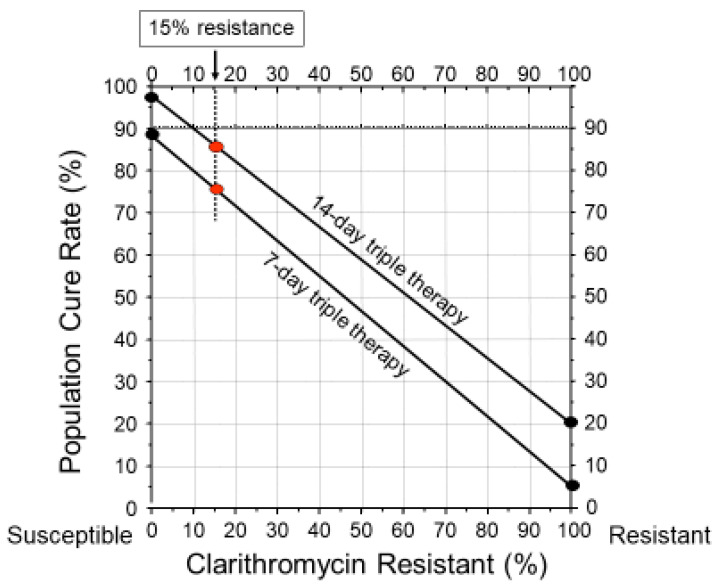
*Helicobacter pylori* treatment nomogram illustrating the duration-related difference in cure rate with 7- and 14-day clarithromycin triple therapy and the effect of clarithromycin resistance on the outcome. As shown, 15% clarithromycin resistance results in a decline in the population cure rate to approximately 85% with 14-day therapy and to approximately 75% with 7-day therapy. It also shows the lack of utility of consensus conference recommendations to use 15% resistance as a yes-no guide to therapy.

With many regimens, particularly with amoxicillin-containing triple therapies, one can predict the population cure rate based on the prevalence of resistance or vice versa. For example, since amoxicillin resistance is currently very rare, it can generally be ignored. In contrast, clarithromycin resistance is all-or-none and clarithromycin is functionally removed from the regimen making the cure rate dependent on the remaining amoxicillin PPI dual therapy [55]. The cure rate for any population can be estimated as follows: (cure rate with susceptible infections x the proportion with susceptible infections) + (cure rate with resistant infections x the proportion with clarithromycin resistance). An alternate approach would be to use an *H. pylori* treatment nomogram (Figure 2) [55]. The nomogram has the advantage of allowing one to easily visualize the effect of the relation of prevalence of resistance on cure rates with different durations of therapy.

Although 14-day therapy may prove to be optimal, clinicians may still be obliged to use a shorter, government-approved duration which may be less effective. Clinicians may also be confused by the recommendations from consensus conferences which, until recently, recommended 7-day triple therapy or, in other instances, recommended a range of durations such as 7 to 14 days. Optimal duration can never be expressed as a range. In addition, consensus recommendations often fail to include the caveats needed to understand when any specific duration would be recommended. The reasons for this lack of clarity are unclear. Possibilities include not wishing to appear opposed to what is approved locally, or bias related to one or more conference sponsors.

Bismuth quadruple therapy does not contain amoxicillin and the antimicrobials used are relatively acid insensitive, such that the lessons learned with amoxicillin-containing therapies may not apply. As noted above, early studies showed that, with metronidazole susceptible infections, a duration of 4 to 7 days was sufficient to achieve cure rates of >95% [56,57,58,59]. However, consensus conferences have almost exclusively recommended 14-day bismuth quadruple therapy [60,61,62,63]. This recommendation was based on the fact that, in many areas, metronidazole resistance is common and increasing, and susceptibility testing is rare. Thus, as a general rule, unless proven otherwise, resistance should be considered to be present which requires one to lengthen the duration of therapy and increase the dosage of metronidazole [13]. In areas where metronidazole resistance is rare or, when metronidazole susceptibility has been confirmed, durations shorter than 14 days are effective and 7-day therapy is generally recommended.

The approval of Pylera^®^ in Europe resulted in an effort to shorten the recommended duration to 10 days to coincide with the approved and marketed duration. In the USA, the duration issue was not a problem, as Pylera^®^ was offered in bottles of capsules which allowed the physician to prescribe, and the pharmacist to dispense, whatever the physician decided was the best duration of therapy for the individual patient. At the same time that tetracycline became very difficult to obtain, the company changed the packaging of Pylera^®^ from bottles to a 10-day dose pack. This requires purchasing two packs to achieve a 14-day therapy for treatment of patients with metronidazole resistant infections, which represents a problem in the USA because the average retail price of Pylera^®^ is $1110/10-day dose pack.

The optimum duration of bismuth quadruple therapy remains unknown, largely untested, and is impossible to prove without head-to-head comparisons in populations where the pattern of resistance is known. Another problem related to doing comparisons is that whether a strain is considered resistant or susceptible to metronidazole may depend in part on the test used. It is unclear why Etest may overestimate metronidazole resistance as compared with results obtained with agar dilution [64,65]. Etest is widely used because it is easier to perform. Because of Etest’s tendency to overestimate the prevalence of metronidazole resistant infections, it has been our practice to always confirm metronidazole resistant results by agar dilution which correlates better with clinical outcome. Efficacy studies relying on Etest to determine metronidazole resistance are more likely to overestimate the therapies’ efficacy in the presence of resistant strains. There are a number of studies ostensibly done to test whether 10-day bismuth quadruple therapy is highly effective. These have been studied in populations with an unknown, but generally low, prevalence of metronidazole resistance [66]. Such studies often achieve cure rates between 88% and 92% which appear suboptimal [8,66,67]. Studies are needed that are designed using the principles of antimicrobial stewardship and all treatment components, including the antisecretory activity of the PPI chosen, antimicrobial dosages, frequency of administration, administration in relation to meals, and the duration of therapy. The question “What is the optimal duration for bismuth quadruple therapy with susceptible infections, with resistant infections, and for populations where the resistant pattern is unknown?” remains unanswered. It is likely that, for susceptible infections, 10 and 14 days are too long and, for resistant infections, both may be too short.

## 11. Poly-Antimicrobial Therapies

The discovery of *H. pylori* and the search for effective treatment coincided with the problem of increasing global antimicrobial resistance. The worldwide increase in macrolide resistance resulted in a precipitous decline in *H. pylori* cure rates with clarithromycin-containing therapies. One response was to modify the current empiric regimens by increasing the number of antimicrobials used (i.e., if two antimicrobials were no longer effective, why not add a third or a fourth?) (reviewed in [30]). This led to the use of a variety of empirically administered regimens containing combinations of a PPI, amoxicillin, clarithromycin, and metronidazole named sequential, concomitant, hybrid, and reverse hybrid therapies. As noted above, 10-day sequential therapy consists of a five-day course of a PPI plus amoxicillin followed by five-day course of a PPI, clarithromycin, and metronidazole. Although the 14-day therapy was more effective, the longer duration has not been widely used, as overall, the regimen has been considered to be obsolete [52].

The alternative was to give all four drugs concomitantly as a concomitant therapy, and proceeded or followed by a dual PPI-amoxicillin therapy (as a hybrid or reverse hybrid therapies). Concomitant therapy is representative of the group. It is functionally equivalent to giving both metronidazole and clarithromycin triple therapies simultaneously [68,69], with success being dependent on the infection being susceptible to amoxicillin and clarithromycin or to metronidazole. Treatment failure requires resistance to both metronidazole and clarithromycin. Although effective, the potential for these three antimicrobial-containing therapies to contribute to the global problem of antimicrobial misuse was not considered. The problem is that all subjects receive at least one antimicrobial not required to cure the infection and whose only function is to potentially contribute to the global antimicrobial resistance (Table 3) [4,69].

The quantity of unnecessary antibiotic misuse with these therapies is not trivial. For example, successful therapy with 14-day concomitant therapy containing 1 gm of metronidazole and clarithromycin would produce 14,000 kg of unneeded antibiotic per 1 million successful treatments and 28,000 kg per 1 million treatment failures. Empiric concomitant therapy was recommended by the Maastricht V, Toronto, and American College of Gastroenterology guidelines [33,61,62], but not by the Houston consensus which considered concomitant therapy to be obsolete [60]. The tendency to add antimicrobials has continued with new sequential therapies and even therapies containing four antimicrobials [30,70]

## 12. Requirements and Impediments for Transition of *H. pylori* Therapy to the Principles of Antimicrobial Stewardship

Although susceptibility testing for human pathogens is available in most hospitals and clinics worldwide, local susceptibility testing for *H. pylori* is almost universally unavailable. In the United States, culture and susceptibility testing for *H. pylori* is currently available from the Mayo Clinic laboratory and a few other major commercial laboratories. However, the details of how to obtain this service remain the responsibility of the individual physician or endoscopy unit. Molecular susceptibility testing of biopsies or stool specimens is also available commercially from a few sources (e.g., American Molecular Laboratories, http://amlaboratories.com/clinical-lab-menu/amhpr-h-pylori-antibiotic-resistance-panel). Consensus statements have typically recommended susceptibility testing only for patients with at least two treatment failures. The reasons why susceptibility testing is lacking are many and include lack of demand, difficulties with reimbursement, lack of a tradition of susceptibility-based therapy to treat *H. pylori,* and lack of surveillance programs to provide local or regional resistance patterns and to guide therapy. This may change in the USA as the Centers for Medicare and Medicaid Services (CMS) has recently published a regulation requiring all hospitals participating in its programs to establish antimicrobial stewardship programs [71]. They require the appointment of a physician and a pharmacist to be responsible for developing plans and procedures to ensure appropriate therapy. Their requirements also include providing susceptibility testing, treatment guides, as well as monitoring of therapy and prescriptions which are included in the U.S. Centers for Disease Control (CDC) guidelines [72]. These include creation and promotion of susceptibility-based treatments, tracking of antibiotic dispensing, and setting targets for improvement (i.e., monitoring and reporting). It is not yet clear whether *H. pylori* infections are included in the mandate.

The current *H. pylori* treatment guidelines have proven to be ineffective, as they have failed to provide recommendations to reliably yield high cure rates and for creation and promotion of susceptibility-based treatments, tracking of antibiotic dispensing, as well as setting targets for improvement [34]. The prevalent A vs. B comparison mentality has been concerned with differences in (a) actual results, (b) rather than whether either achieved acceptable cure rates, or (c) for understanding the reason for the differences (i.e., the data required to reliably achieve high cure rates). As noted above, most meta-analyses have involved studies whose results are only relevant to the individual study included and are not generalizable or useful for any other population [30].

Consensus conferences and guidelines have often failed to provide clinically useful guidance regarding therapy. For example, as noted above, by 2001 empiric clarithromycin triple therapy was proven to no longer achieve clinically acceptable cure rates in most areas. Rather than state outright that clarithromycin should no longer be used empirically, the 2006 Maastricht III conference suggested using a cutoff of 15–20% resistance above which clarithromycin should not be used empirically [73]. They noted, “Clarithromycin resistance is increasing. It is the main risk factor for treatment failure. Treatment should achieve an eradication rate of >80%. The threshold of clarithromycin resistance at which this antibiotic should not be used, or a clarithromycin susceptibility test should be performed is 15–20%.” The cut-off was further refined to >15% in the 2012 Maastricht IV confirmed in the 2017 Maastricht V consensus [61,74]. In retrospect, these guidelines were both impractical and toothless as they were impossible to implement because the required susceptibility data was unavailable. Subsequent analyses of worldwide *H. pylori* resistance have confirmed that resistance exceeded 15% for clarithromycin, metronidazole, and levofloxacin in all WHO regions [75].

The more recent Houston consensus recommended that clarithromycin, metronidazole, and levofloxacin should not be used empirically unless proven to be reliably highly effective locally [60]. Post treatment test-of-cure is currently the only method that most clinicians can use to indirectly assess susceptibility/resistance patterns. With few exceptions, routine testing for cure has been recommended for decades. As a surveillance tool, the results, if heeded, provide information whether a regimen does, or does not, reliably achieve high cure rates. In theory, this information would be collected, shared, and used to indicate whether a regimen should be replaced or modified. Overall, it appears that while this simple measure may be used to assist in the management of individual patients it has been ineffective as a surveillance tool [8,33]

## 13. Adoption of the Principles of Antimicrobial Stewardship

While the goal is straightforward, accomplishing adoption of antimicrobial stewardship will not be easy as it requires a major paradigm shift (Table 4) [32].

One of the first steps is to develop treatment guidelines based on the principles of antimicrobial stewardship. All recommendations not based on antimicrobial stewardship should be replaced by ones proven to reliably produce high local cure rates. To date, none of the currently used or recommended treatment regimens have been optimized nor do they consider local resistance patterns. In addition to providing new treatment guidelines, surveillance of treatment outcomes must routinely be monitored to assure continuing effectiveness. Clinical trials should focus on achieving high cure rates (e.g., >95%). “Good enough” is not good enough. Comparative trials should be restricted to comparisons of proven highly effective therapies that utilize noninferiority methodology. Studies using a regimen known to have an inferior cure rate as a comparator without truly informed consent are unethical and should not be done and, if done, should not be published [25,32] ( Table 2; Table 4). No regimen should be used empirically unless it has been proven to reliably achieve high cure rates in the target population. Surveillance programs should be implemented to provide early warning if the effectiveness of currently recommended therapies declines, so that new therapies and guidelines can be implemented. Until susceptibility testing becomes widespread, surveillance should consist of routine tests-of-cure and the results should be reported in order to alert clinicians when a regimen no longer should be prescribed empirically. Current ongoing local and regional antimicrobial surveillance programs should include *H. pylori.* Large effective consortia, such as the European Registry on *Helicobacter pylori* Management, should be repurposed from simply collecting treatment results to providing surveillance, susceptibility testing, and up-to-date treatment recommendations. Clearly, we still have a long way to go and many things to do to adopt antimicrobial stewardship for *H. pylori* therapy.

## 14. Proposal Regarding How to Improve Empiric Therapies While Introducing Antimicrobial Stewardship

Fundamentally, the goal is to reliably achieve high cure rates in routine clinical practice (Table 5). The principles are as follows: First, to use only regimens proven to achieve high cure rates locally; second, to provide real-time information about whether the goal is achieved by routinely monitoring and reporting outcomes based on test-of-cure data; third, to abandon or modify therapies that fail to reliably achieve the desired high cure rates.

Clinically, cure is defined using a noninvasive test such as a negative urea breath test at least four weeks after ending therapy or a negative stool antigen test preferably at least six weeks post therapy. Defining what is the minimal high cure rate is complicated by the fact that these noninvasive tests are only approximately 95% sensitive and specific. This limitation is overcome in clinical trials by requiring two positive or negative tests using different methodology (e.g., histology and UBT). This is not practical in daily practice and the cure rate that best approximates ≥95% needs to be identified experimentally. As noted above, the sensitivity and specificity of the noninvasive tests currently used to determine cure are at best 95%. Currently, only one test, typically the UBT, is used and it is practically impossible to reliably confirm that ≥95% have been cured. As such, we propose the cut-off of ≥93% until the testing required to define cure allows a more precise estimate. For population-based clinical results, the cure rate should be based on modified intention-to-treat results that include only those who have test-of-cure data. Obtaining a test-of-cure should be vigorously attempted on all patients irrespective of the duration of therapy (e.g., even for one day), as well as those on lost to follow-up for weeks or even months.

All locally or regionally approved therapies should be proven to reliably achieve high cure rates. These recommendations should include the following: (a) antibiotic doses and frequency of administration; (b) PPI minimum dosage should be 60 mg omeprazole equivalent (e.g., 60 mg omeprazole, 60 mg lansoprazole, 40 mg esomeprazole or rabeprazole, or vonoprazan 20 mg); (c) twice daily for 14 days; and (d) the duration should be 14 days unless the regimen has been formally optimized to use a different duration (Table 6).

Therapies that contain unneeded antibiotics should not be prescribed (e.g., those with three antibiotics, such as concomitant or sequential therapies, or vonoprazan triple therapies) [76]. The test-of-cure result should also be used as part of ongoing surveillance. Ideally, the results should be reported to a central site so that data from an area/region can be pooled and shared.

Regimens that fail to achieve the prespecified endpoint in a prespecified percentage (e.g., 10%) should be removed from the list of approved empiric therapies, although they may remain on the list of approved susceptibility-based therapies.

Although all the approved therapies are expected to achieve high cure rates (i.e., they have been quasi-optimized), each should be formally optimized, as they most likely canfurther improved. Optimization of therapies should be one of the first goals in the introduction of antimicrobial stewardship. As noted above, any modification of an approved therapy should first be confirmed as reliably highly effective using pilot studies without a comparator that also include susceptibility testing. A head-to-head comparison using noninferiority methodology should only be considered after a new or modified regimen has been proven to achieve a high cure rate.

## Figures and Tables

**Table 1:** Definitions of terms to describe outcome of therapy.

Term	Definition
Successful	Excellent or good results
Excellent results	Reliably achieve 95% or greater cure rates in adherent patients with susceptible infections
Good results	Reliably achieve 90% or greater cure rates in adherent patients with susceptible infections
Optimum duration	Days of therapy required to reliably achieve good to excellent results
Doses and frequency of administration	Those that will reliably achieve good to excellent results

From [4], with permission.

**Table 2:** Guidelines to implement antimicrobial stewardship for treatment of *H. pylori* infections.

Therapies must be optimized to reliably achieve high cure rates. Optimization should include the effects of resistance to the different components. Preferably, optimization should be confirmed in different regions.
Surveillance programs should be instituted. At a minimum, this should include tests of cure and, preferably, with susceptibility testing available for treatment failures.
Treatment of *H. pylori* should be integrated with ongoing or planned prescription and treatment monitoring utilized for other bacterial infections.
Data from sites where culture and susceptibility testing and/or molecular testing are done locally should be published and kept up to date.
Susceptibility testing should be reimbursed as for other bacterial pathogens and the results data should be submitted to local and central repositories responsible for monitoring resistance among bacterial pathogens.
To avoid unethical studies, studies should adhere to the guidelines of the Infectious Diseases Society of America regarding conduct of superiority and organism-specific clinical trials of antibacterial agents for the treatment of infections caused by drug-resistant bacterial pathogens.

Adapted from [32].

**Table 3:** Hypothetical scenario of number of unnecessary antibiotics given in relation to antibiotic susceptibility patterns.

Sensitivity Pattern of *H. pylori* to Clarithromycin and Metronidazole		Prevalence of Pattern	Successful Treatment of *H. pylori*	Number of Ineffective Drugs Used	Number of Unnecessary Drugs Used
Clarithromyin: Susceptible 80%; Resistant 20%	Metronidazole: Susceptible 60%; Resistant 40%				
Susceptible	Susceptible	48%	Yes	0	1
Susceptible	Resistant	32%	Yes	1	1
Resistant	Susceptible	12%	Yes	1	1
Resistant	Resistant	8%	No	2	2

Legend: Table showing the number of ineffective or unnecessary antibiotics used by a population of patients similar to those seen in Texas with the *H. pylori* resistance pattern of 20% resistant to clarithromycin, 40% resistant to metronidazole (8% dual resistance) which receives concomitant therapy with a PPI, amoxicillin, clarithromycin, and metronidazole. From [69] with permission.

**Table 4:** American Society of Infectious Diseases criteria for ethical active-controlled superiority studies of antibacterial agents.

The control (i.e., the comparator drug) is active against most, or all, of the bacterial strains likely to be encountered in the study;
2. All available drugs that could be used as comparators for the study are inadequately active against the strains likely to be encountered, such that there is no alternative effective therapy possible; or
3. The infection under study is almost universally non-fatal, such that rescue therapy can be instituted rapidly enough to preclude serious sequelae upon recognition that the strain causing the infection is resistant to the comparator drug (e.g., uncomplicated urinary tract infection). The susceptibility of etiologic bacteria is almost never known at the time an infected patient is enrolled in a clinical trial that evaluates initial antimicrobial treatment. Therefore, the comparator drugs chosen for study in antibacterial clinical trials are selected because they are anticipated to be effective against all, or almost all, strains likely to be encountered during conduct of the study.

Adapted from reference [32].

**Table 5:** Reliable achievement of high cure rates with empiric therapies in clinical practice.

**Principle 1** Only regimens proven to reliably achieve high cure rates.
**Principle 2** Routinely monitor and report outcomes using test-of-cure result to provide real-time information about whether the goals are being met.
**Principle 3** Abandon or modify the therapies that fail to reliably achieve the desired high cure rates.

**Table 6:** Elements of empiric regimens used while the principles of antimicrobial stewardship are being introduced.

Antibiotic doses and frequency of administration should be identified experimentally.
The duration should be 14 days, unless the regimen has been formally optimized to use a different duration.
PPI minimum dosage should be 60 mg omeprazole or equivalent (e.g., 60 mg omeprazole, 60 mg lansoprazole, 40 mg esomeprazole or rabeprazole), or 20 mg vonoprazan given twice daily.
